# Perioperative care of a patient with immune thrombocytopenia purpura undergoing tubo‐ovarian abscess incision and drainage: Case report

**DOI:** 10.1002/ccr3.9534

**Published:** 2024-11-17

**Authors:** Liqiong Zeng, Libi Tian

**Affiliations:** ^1^ University‐Town Hospital of Chongqing Medical University Chongqing China

**Keywords:** bleeding events, clinical challenges, immune thrombocytopenia purpura, tuba‐ovarian abscess

## Abstract

**Key Clinical Massage:**

Tubo‐ovarian abscess (TOA) is a serious health hazard for women, causing severe sepsis. Antimicrobial treatment is effective, but one‐third of patients experience unfavorable outcomes. ITP, an autoimmune condition, can lead to bruising and bleeding. Diagnosing TOA in women of childbearing age is crucial, and combining emergency surgery with ITP patients can increase treatment costs and reduce quality of life. ITP can lead to severe complications, including postoperative hemorrhage, and may require platelet transfusions, glucocorticosteroids, and immunoglobulin. These treatments increase costs, decrease quality of life, and impact prognosis. Preventing ITP is crucial. Patients should be administered blood products based on platelet count and anemia or spontaneous bleeding tendencies. Perioperative blood management should aim for a target platelet level of 30 × 10^9^/L and a hemoglobin concentration of 80 g/L before surgery. Post‐surgery, perioperative care is crucial and vigilant for secondary bleeding.

**Abstract:**

A tubo‐ovarian abscess (TOA) is a frequently encountered inflammatory mass in therapeutic settings. TOA is a serious consequence of pelvic inflammatory disease (PID) that can lead to severe sepsis. In recent years, the incidence of TOA has increased, presenting a significant health hazard for women. To effectively target the diverse range of bacteria responsible for TOA, it is essential to use antimicrobial medicines that have a wide spectrum of activity. Nevertheless, the efficacy of antibiotic treatment stands at approximately 70%, while a significant proportion of patients, around one‐third, experience unfavorable clinical outcomes necessitating drainage or surgical intervention. Immune thrombocytopenia (ITP) is an autoimmune condition characterized by a marked decrease in the quantity of platelets present in the bloodstream. ITP is characterized by thrombocytopenia, which leads to a heightened susceptibility to bruising and bleeding. The diagnosis of ITP and the prediction of treatment response continue to pose important and persistent issues in the field of hematology. The platelet count is commonly employed as a surrogate indicator of disease severity in patients with ITP and thus plays a crucial role in determining the necessity of treatment. A 25‐year‐old woman with a history of sexual activity underwent open abdominal exploration due to the sudden onset of lower abdominal pain. During the operation, a left TOA was discovered, and an incision and drainage were performed. Symptomatic treatments, such as anti‐infectives and abdominal drainage, were administered. The culture of pus in the abdominal cavity suggested the presence of Escherichia coli. However, the patient presented with ITP and had a platelet count of less than 50 × 10^9^/L before the operation. After the operation, the patient developed incisional and pelvic hematomas with signs of infection. As a result, the patient was discharged from the hospital after undergoing another laparotomy and receiving platelet transfusions and immunotherapy. Clinicians should be vigilant when diagnosing TOA in women of childbearing age, even in the absence of high‐risk factors. A timely antibiotic or surgical intervention is necessary to preserve fertility and ensure quality of life. Combining emergency surgery with ITP patients poses a significant challenge for clinicians in terms of treatment. ITP can lead to serious complications, such as postoperative bleeding, which may require platelet transfusions, glucocorticoids, and immunoglobulin. This can increase the cost of treatment, reduce the quality of life, and seriously affect the prognosis. Therefore, preventing ITP is crucial. It is important to pay attention to the perioperative care of patients after surgery and be alert to the possibility of secondary hemorrhage.

## 1 INTRODUCTION

TOA is a well‐known and severe consequence of untreated PID. The condition primarily impacts women in their reproductive years, and around 60% of women diagnosed with TOA have never given birth.^1^ TOA, or tubo‐ovarian abscess, is a condition marked by an inflammatory mass that affects the fallopian tube and/or ovary and is characterized by the presence of pus.^2^ TOA is a type of infection caused by a combination of anaerobic, aerobic, and facultative aerobic bacteria.^3^ Primarily observed in females within the reproductive age range, The primary risk factors include engaging in sexual activity with several partners, having a history of PID, and having a weakened immune system.^4^ While the clinical presentation may differ, common symptoms include fever, stomach pain, and complaints of vaginal discharge. The diagnosis is established when the clinical observations are accompanied by elevated inflammatory markers and radiographic evidence revealing the presence of a mass. Due to the potential life‐threatening complications of TOA, such as the danger of rupture and sepsis, prompt action is necessary from the moment of diagnosis to treatment. First‐line treatment typically involves the use of broad‐spectrum antibiotics. Multiple studies have demonstrated that antibiotic treatment has an effectiveness ranging from 34% to 87.5%.^5^ However, surgical intervention is necessary for patients when medicinal treatment becomes inadequate and there is an abscess rupture. Surgical intervention may be necessary, but the ideal timing and the most suitable treatment remain uncertain. Techniques encompass laparoscopic surgery versus open surgery and the choice between draining an abscess or doing a drastic excision. Despite a dramatic reduction in mortality rates in the treatment of TOA during the past 50 years, there are still associated complications such as late infertility, ectopic pregnancy, persistent pelvic pain, and ovarian vein thrombosis.^6^

ITP is an autoimmune disorder marked by heightened platelet destruction and inhibition of creation, leading to a specific condition of low platelet count.^7^ ITP is defined by thrombocytopenia (PC; <100 × 10^9^ platelets/L) and an elevated susceptibility to bleeding.^8^, ^9^ In adult patients, notable clinical symptoms include bleeding problems, hemorrhages in the skin or mucous membranes, including purpura and petechiae, and infrequently, signs of the disease in the cerebral region.^10^ Treatment methods for ITP are primarily determined by the clinical symptoms of patients, with an emphasis on minimizing the likelihood of significant bleeding rather than specifically aiming to increase platelet counts. According to the guidelines set by the International Working Group,^11^ patients who have acute ITP and do not have a history indicating a high risk of severe bleeding should be treated using an observation strategy, also known as a “wait and see” approach. However, patients with ITP necessitate immediate medical intervention if they are susceptible to an elevated risk of hemorrhaging or have a severe instance of chronic thrombocytopenia. Furthermore, it is crucial to tailor treatment regimens according to the specific requirements and risk profiles of each patient.^12^, ^13^

We present a case study of a 25‐year‐old woman with a history of sexual activity and primary ITP. The patient underwent emergency incision and drainage of a left TOA and experienced postoperative complications, including incision hematoma with infection and pelvic hematoma formation. The patient was readmitted for further investigation and discharged after receiving platelet transfusions and immunotherapy. In the case of women with tubo‐ovarian abscesses, it is important to intervene in treatment to ensure patient fertility and quality of life. Prevention of ITP is crucial as it may lead to serious complications, such as bleeding after surgery. Therefore, perioperative care should be taken, and the possibility of secondary bleeding should be monitored.

## 2 CASE PRESENTATION

### 2.1 Case history/examination

A 25‐year‐old unmarried woman who is infertile and has a history of sexual intercourse was admitted to the hospital with lower abdominal pain that had been worsening for 7 h over the course of 1 day. The patient had a history of skin ecchymosis and petechiae since childhood. They were diagnosed with untreated immune thrombocytopenic purpura at an outside hospital 6 years ago, with platelets fluctuating between 50 and 80 × 10^9^/L at irregular follow‐up visits. The patient's mother, grandmother, and maternal uncle also suffer from ITP.

The patient experienced vague lower abdominal pain with diarrhea without an obvious cause 1 day before admission. Seven hours before admission, the pain worsened without any apparent trigger, and the patient experienced paroxysmal colic and unbearable pain with no obvious relief position. The patient also experienced nausea, vomiting, and anal distention in the outpatient clinic. The patient tested negative for HCG in the urine. Blood tests revealed leukocytes at 22.2 × 10^9^/L, neutrophils at 70.4%, and platelets at 56 × 10^9^/L. The gynecological ultrasound showed increased bright spots in the uterine cavity and a cystic lesion in the left adnexal region (approximately 2.5 × 1.9 cm).

**FIGURE 1:**
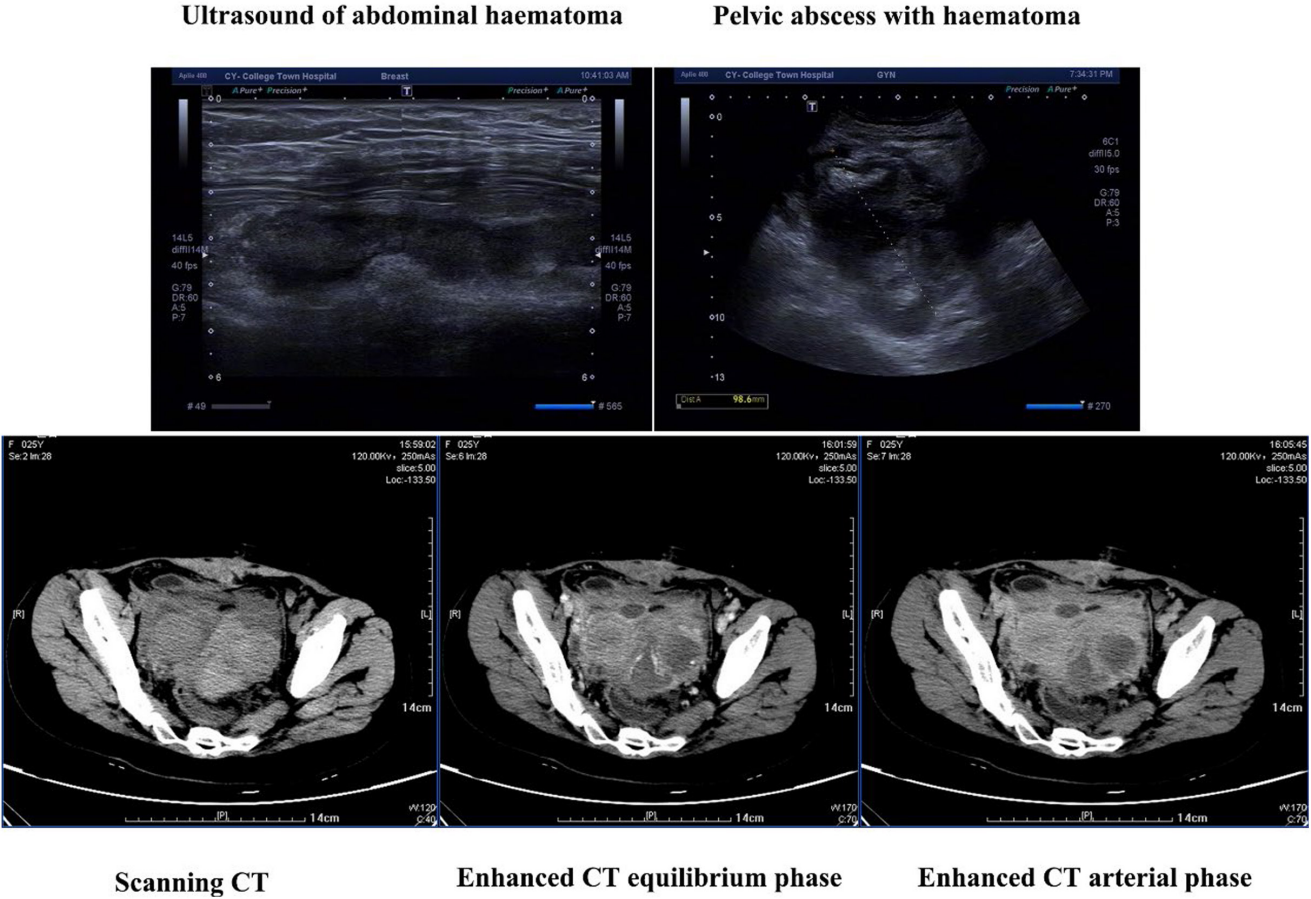
Pelvic ultrasound (abdominal wall haematoma, pelvic haematoma with abscess) and CT images (pelvic haematoma with abscess).

A patient was evaluated in the emergency department of a hospital, and a follow‐up ultrasound revealed an abnormally echogenic area in the left adnexal region. The internal echogenicity was chaotic, with some poorly translucent anechoic and slightly high echogenicity. The CT scan of the abdomen revealed a mixed‐density mass shadow in the left adnexal area, with the larger level measuring approximately 63 × 56 mm. The nature of the mass is yet to be determined, and there is a possibility of peritonitis (Figure 1). The patient's blood routine showed a white blood cell count of 19, a neutrophil percentage of 88.4%, and approximately 1000 blood clots, with one located in the left mid‐abdomen. The patient experienced chills, fever, dizziness, and malaise during the consultation. His blood pressure dropped as low as 87/57 mmHg, and he was treated with venous access, indwelling catheterization, rehydration fluids, and dobutamine to increase blood pressure. The medical department recommended hospitalization for surgical treatment. The patient was admitted to the emergency department to determine if he had left ovarian tumor apex torsion or right ovarian corpus luteum rupture causing lower abdominal pain.

### 2.2 Methods

Following admission, preoperative preparation and patient communication were optimized, and an emergency caesarean section was performed under general anesthesia. During the operation, approximately 300 mL of yellowish‐white pus was observed in the abdominal cavity, confirming the diagnosis of a left TOA. The abscess was subsequently drained during the operation. Postoperatively, the patient received symptomatic supportive therapy, including anti‐infection treatment, hemostasis, albumin infusion, and abdominal drainage. A culture of the abdominal pus tested positive for *Escherichia coli*. Humoral immunity, autoimmunity, and ANCA were normal. The post‐operative examination revealed left tubal congestion, hemorrhage, and oedema with acute and chronic inflammatory cell infiltration. Organized abdominal adherent tissue Inflammatory cell infiltration and fibrin‐like material were observed in massive blood clots.

Following an operation, the patient experienced intermittent fever, coughing, and sputum. On the third day, a chest CT revealed bilateral pleural effusion and mild external compressive atelectasis in both lower lungs. A respiratory consultation diagnosed acute bronchitis, and antibiotics were added to continue anti‐infective and cough treatment. The patient experienced postoperative platelet fluctuation ranging from 27 to 30 × 10^9^/L. On the 10th day, the patient still had a high fever. The patient's skin exhibited both new and old petechiae, primarily on the limbs. The lower abdomen was slightly distended with a slightly high tone, and Petechiae were observed around the mid‐abdominal incision. A blood test revealed a white blood cell count of 15.67 × 10^9^/L, hemoglobin level of 101 g/L, and platelet count of 29 × 10^9^/L. The neutrophil percentage is 88.3%, and the C‐reactive protein level is 49.40 mg/L. The pelvic enhancement CT reveals a mixed‐density mass shadow in the left adnexal area, indicating possible bleeding within the lesion. The patient may have experienced luteal cyst rupture bleeding. The current film shows a more severe case of diffuse peritonitis with pelvic effusion compared to the previous one. Additionally, a submuscular abnormal density shadow is located in the middle of the anterior lower abdominal wall. The abdominal incision ultrasound shows abnormal echoes in the deep surface of the muscle layer of the abdominal wall at the incision site, suggesting a hematoma may form. The ultrasound report also raises the question of abscess formation.

### 2.3 Conclusion and results

**FIGURE 2:**
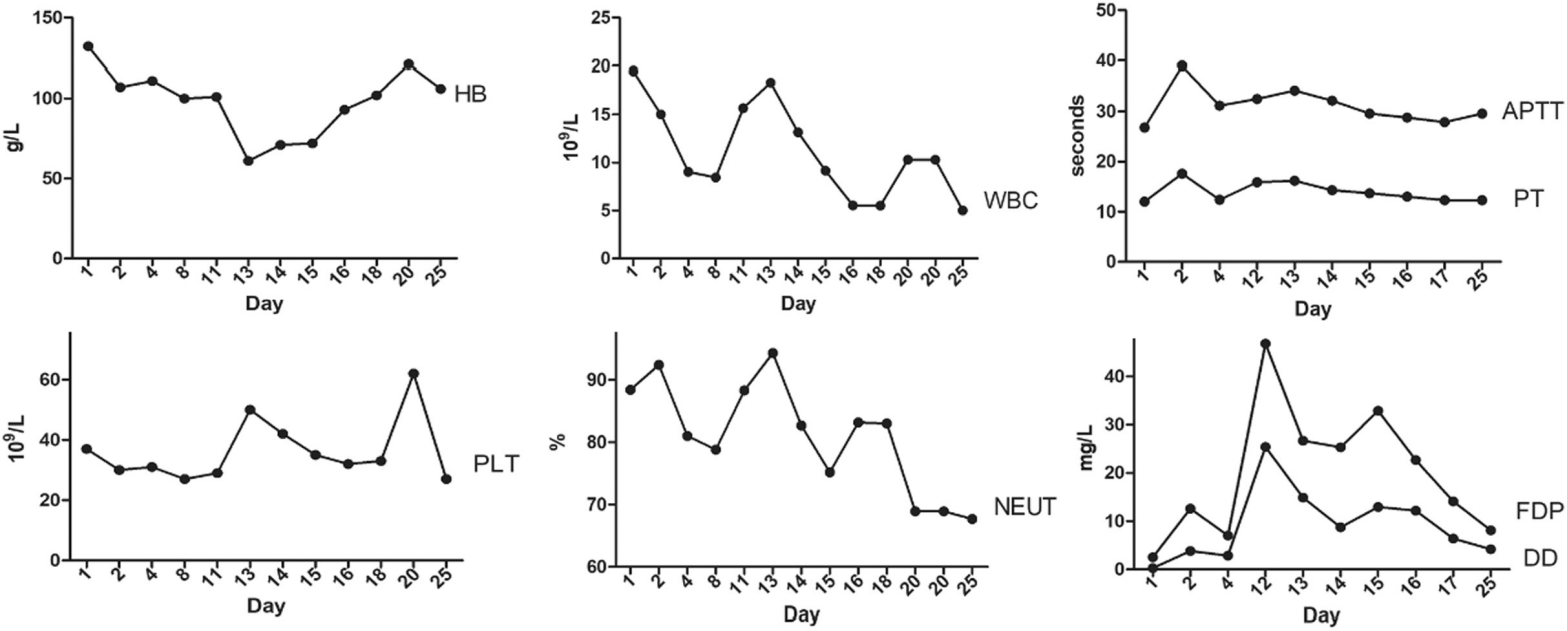
Curve of changes in hematological indicators (hemoglobin, platelets, white blood cells, neutrophil percentage, prothrombin time, partially activated prothrombin time, D‐dimer, fibrinogen) during hospitalization.

A patient was diagnosed with an incisional hematoma, infection, and pelvic abscess. Urgent surgery was performed on the 11th day, involving a caesarean section, left salpingo‐oophorectomy, and debridement of the pelvic hematoma and abdominal wall incision hematoma. The surgery was successful, but the patient experienced intraoperative bleeding of 200 mL and an additional 800 mL of blood and clot accumulation in the peritoneal cavity. The patient's vital signs remained stable during and after the operation. They were transferred to the ICU for further treatment, anti‐infection treatment, hemostasis, blood transfusion, albumin, and other supportive care. On the third day, they were transferred back to the department for further treatment. The patient's blood routine was followed, and platelet fluctuation was observed to be 27–62 × 10^9^/L. The pelvic drainage tube was removed on the 6th and 8th days, and the abdominal incision healed well after the operation. A blood test was repeated on the 14th day, revealing a hemoglobin level of 106 g/L and a platelet count of 27 × 10^9^/L (Figure 2). The patient requested discharge from the hospital, but no new onset or active bleeding was observed.

## 3 DISCUSSION

PID typically arises from an infection that spreads upwards, affecting various parts of the reproductive system such as the uterus (endometritis and myometritis), fallopian tubes (salpingitis), ovary (oophoritis), wide ligaments (parametritis), and abdomen (peritonitis).^14^ If not treated, PID can deteriorate and result in the development of an abscess, medically referred to as a TOA.^15^ TOA is a severe complication of PID, as it has the potential to extend beyond the boundaries of the upper genital tract and affect other pelvic organs.^4^ TOA are more likely to occur in individuals who have PID or a previous sexually transmitted infection (STI), previous surgery in the abdominal or pelvic area, anatomical abnormalities in the genital tract, a male partner with an active STI, and those who have undergone fertility treatments, especially if egg retrieval was performed.^16^, ^17^ The cause of the condition is typically attributed to many microorganisms. The microorganisms commonly found in TOAs are *E. coli*, Streptococci, Bacteroides fragilis, Prevotella, and other gramme‐negative enteric organisms.^6^

Acute TOA is a complex condition characterized by a variety of symptoms and indications. These include adnexal tenderness, cervical excitation, fever, abnormal discharge, increased white blood cell count, elevated erythrocyte sedimentation rate, C‐reactive protein, positive test results for Neisseria gonorrhoeae and/or Chlamydia trachomatis, and detection of an adnexal mass. In severe cases, other signs of systemic sepsis may also be observed. TOA patients are more likely to experience fever and diarrhea compared to those with PID, with rates of 90% and 60%, respectively.^18^ Alternative diagnoses include appendicular tumors, endometrioma, ectopic pregnancy, diverticulitis, or underlying cancer. The inflammatory activity within the pelvis may also be confined to neighboring structures like the omentum and bowel.^19^

Pelvic ultrasonography is the primary imaging modality.^20^ Based on the literature, transvaginal ultrasound has a greater level of accuracy compared to trans‐abdominal ultrasonography.^20^ The sonographic presentation of TOA is distinguished by the presence of complex multilocular cystic masses with thick and uneven walls, mixed echogenicity, and distortion of the typical anatomical features.^21^ Additional imaging modalities frequently utilized include computed tomography (CT) and magnetic resonance imaging (MRI), which have shown good outcomes in the identification of TOA. Clinically, computed tomography or magnetic resonance imaging are often used to make a more certain diagnosis when treating TOA that does not respond to antibiotics or when sonographic results are not clear. Typical radiographic characteristics of TOA include thick‐walled cystic masses in the adnexa, which have internal divisions and are accompanied by inflammatory alterations in the surrounding area.^22^, ^23^, ^24^ The primary observations in the MRI scans reveal the existence of a pelvic mass exhibiting reduced signal intensity on T1‐weighted images and varied high signal intensity on T2‐weighted images.^24^

Treatment of TOA should involve the use of broad‐spectrum antibiotics. The goal of antibiotic treatment is to achieve a balance between fluctuating levels of antibiotics in the bloodstream and effectively eliminating bacteria while also minimizing any negative effects. Inflammatory indicators, which indicate the presence of inflammation, can be utilized during the inspection, monitoring, and assessment of therapy efficacy. The efficacy of antibiotics as a standalone treatment ranges from 34% to 87.5%.^21^ Simultaneously, the frequency of recurrence is greater in individuals who are solely treated with antibiotics. It is crucial to note that the antibiotics used should include Bacteroides fragilis, Peptostrepto‐coccus, gramme‐negative aerobes, Neisseria gonorrhoeae, and Chlamydia trachomatis in order to address the cause of TOA. There are treatment regimens for this condition that involve the use of two or three therapies simultaneously.^25^

The woman with a TOA needs acute management, including monitoring her vital signs using a standardized early warning system chart. Fluid balance and urine output should be closely monitored, with a urinary catheter being used for accurate evaluation. Blood indicators, such as leukocyte count and C‐reactive protein levels, should be monitored daily. Prophylaxis against venous thromboembolism should involve compression stockings, and if surgery is not expected, low‐molecular‐weight heparin should be explored. A minimum of two evaluations by a senior doctor within 24 h is recommended. A multidisciplinary approach is expected to yield the best outcomes. If the woman's condition worsens, she may need more medical attention in a high‐dependency or intensive care unit.

In some cases, surgical intervention may be necessary due to the high prevalence of Toxic Abscess Asthma (TOA) in women of childbearing age. The mortality rate for TOAs that have not burst is low, but it can range from 1.7% to 3.7% in cases where TOA rupture occurs.^1^ The surgical procedure for TOA can be laparoscopy, laparotomy with abscess drainage, unilateral or bilateral salpingo‐oophorectomy, or pelvic clearance. The choice of surgical procedure depends on the patient's surgical history, fertility preferences, and the size of the abscess. If the patient meets the criteria for a laparoscopic procedure and the surgeon has adequate laparoscopic abilities, a shorter recovery period is achieved. However, some women may still opt for a midline laparotomy when dealing with TOAs due to prior surgery, a sizable abscess, or a concurrent ailment like inflammatory bowel disease. The surgical procedure for TOAs can pose technical challenges due to the delicate nature of necrotic tissue, which is prone to fragility, tissue collapse, and hemorrhaging. The presence of TOA increases the likelihood of a visceral injury.^18^ Following surgery, antibiotics should be administered through the intravenous route, and a sample of pus should be collected to determine the most appropriate antibiotic treatment. Prolonged fever may require frequent consultations with microbiological colleagues and adjustments in antibiotic choice.

Untreated TOA can result in persistent consequences, including chronic abdominal pain, painful sexual intercourse, inflammation of the abdominal lining, the formation of scar tissue in the abdomen, the spread of infection beyond the pelvic region, a heightened likelihood of ectopic pregnancy, and sterility of the fallopian tubes.^26^ One occurrence of TOA can greatly heighten the likelihood of tubal infertility, and consecutive occurrences of TOA further amplify this risk.^27^

In this case, the patient was a woman of childbearing age with a history of sexual intercourse but no other risk factors for TOA. Upon admission, an abdominal ultrasound and CT scan were performed, which suggested the need for emergency open abdominal exploratory surgery to address either left ovarian tumor tip torsion or rupture of the corpus luteum of the right ovary. However, during the operation, it was discovered that the mass on the left ovary was a TOA. As a result, the abscess was incised and drained, and the patient received postoperative symptomatic treatments, including anti‐infection medication and abdominal drainage. The postoperative culture of pus in the abdominal cavity revealed the presence of *E*. *coli*. The examination indicated congestion, hemorrhage, and oedema in the left fallopian tube, along with acute and chronic inflammatory cell infiltration. A massive blood clot with inflammatory cell infiltration and fibrin‐like material was found in the abdominal adherent mechanized tissue. Although this patient did not have risk factors for TOA, we should remain vigilant for this diagnosis in our clinical work. To ensure the patient's fertility and quality of life, timely surgical intervention and postoperative anti‐infective treatment are both necessary to treat the condition.

ITP is an autoimmune illness characterized by a low platelet count due to heightened platelet breakdown and suppression of platelet synthesis.^9^, ^28^ As per the current recommendations, a diagnosis of ITP can be made when there is a low platelet count (<100 × 10^9^/l) without the appearance of anemia, leukopenia, or any other known causes of thrombocytopenia.^29^ The most reliable proof of an ITP diagnosis in clinical practice is observing a positive response to therapy specifically designed for ITP. The overall prevalence is around 3.3 cases per 100,000 adults per year.^30^

ITP is thought to arise due to abnormalities in immunological tolerance. There are two primary mechanisms that contribute to the development of ITP: enhanced platelet breakdown and inadequate platelet formation. The disease's etiology is intricate and not completely comprehended; however, both antibody‐mediated and/or T cell‐mediated platelet destruction are fundamental mechanisms. Autoantibodies are commonly believed to be generated and attach to glycoproteins found on the surface of platelets. This attachment identifies the platelets for phagocytic breakdown in the spleen and liver by interacting with Fcγ receptors.^9^, ^31^ Furthermore, it has been documented that autoantibodies might enhance the death of platelets by complement‐mediated or desialylation‐induced mechanisms, as well as hinder the generation of new platelets by megakaryocytes.^29^ Nevertheless, a significant proportion of patients with ITP, ranging from 30% to 40%, do not exhibit detectable antibodies, indicating the involvement of other pathways.^9^

Experiencing a hemorrhagic episode has been associated with unfavorable outcomes and heightened mortality in patients with ITP. Bleeding typically encompasses hematoma, purpura, petechiae, oral, and nasal hemorrhages, as well as significant menstrual or urogenital bleeding, and it is normally of mild to moderate intensity. However, it has been reported that intracerebral hemorrhage (ICH) and other types of serious bleeding can occur in approximately 1% and 15% of instances of ITP. ITP patients with platelet counts between 25 and 49 × 10^9^/L and <25 × 10^9^/L had 2.4 and 4.5 times higher bleeding rates, respectively, compared to ITP patients with normal platelet counts.^32^ Interestingly, there have been reports of a higher occurrence of thromboembolic events in individuals with ITP.^33^, ^34^ Hence, it is imperative for individuals with ITP to be cognizant of the potential for thromboembolic events. Patients should be informed that ITP can elevate the likelihood of both bleeding and venous and arterial thromboembolism.^35^ In individuals with ITP, the death rate is slightly greater than that of the general population. This is mainly attributed to elevated rates of cardiovascular disease, infection, bleeding, and mortality attributable to hematological malignancies.^36^

Autoantibodies are crucial in diagnosing isolated thrombocytopenia (ITP), which often leads to exhaustion and minor bleeding.^37^ Platelet count, blood and bone marrow smears are the primary clinical methods for assessing ITP cases. Secondary causes of ITP include screening for autoimmune illnesses, serology tests for hepatitis C and HIV, and Helicobacter pylori in certain regions.^38^, ^39^ Blood samples can be examined using monoclonal antibodies and light microscopy to detect platelet alterations and decreased platelet receptor amounts, helping to exclude macrothrombocytopenia‐related illnesses. Measuring platelet diameter can differentiate ITP from other types of thrombocytopenia, such as myelodysplastic syndromes, drug‐induced thrombocytopenia, hereditary thrombocytopenia, pseudo‐thrombocytopenia, and bone marrow failure syndromes.^40^, ^41^

ITP treatment aims to address acute and severe bleeding and prevent future occurrences. Current management is not strictly standardized, with initial treatment typically involving steroids or IVIG, or a combination of both. Second‐line treatment mainly consists of thrombopoietin receptor agonists (TPO‐RAs) and rituximab. A splenectomy is postponed until 1 year after the first diagnosis. Other second‐line medications include fostamatinib and immunosuppressive agents. There is no standard for the specific sequence of second‐line drugs. The American Society of Hematology recommends TPO‐RAs as the initial choice for patients with ongoing disease. Treatment‐resistant illnesses may require multiple therapeutic drugs, but splenectomy may not be a standard procedure.^41^, ^42^

ITP is a condition characterized by various factors, including platelet count and bleeding severity.^43^ Treatment decisions are influenced by these factors, including bleeding severity, age, existing medical conditions, disease stage and duration, activity level, potential negative effects, and patient‐related factors like well‐being and fatigue. The therapeutic objectives include preventing significant bleeding, maintaining a targeted platelet count of 20–30 × 10^9^/L, minimizing adverse effects, and enhancing quality of life.^13^ Accurately categorizing patients based on bleeding risk is beneficial in invasive operations and perioperative care. The International Consensus Report recommends a platelet count of 50 × 10^9^/L for small procedures and 80 × 10^9^/L for large surgeries.^10^ Platelet transfusions may be administered to ITP patients as part of preoperative therapy to mitigate bleeding complications. Accurately categorizing patients based on bleeding risk is also beneficial in invasive operations and perioperative care.

There are several aspects of ITP that specifically impact women's health and quality of life, including pregnancy, childbirth, postpartum state, menstrual bleeding, and birth control (contraception).^44^, ^45^ In this case, the patient has had a history of skin ecchymosis and petechiae since childhood. She was diagnosed with ITP at an outside hospital 6 years prior to admission. Additionally, her mother, grandmother, and maternal uncle also suffered from ITP. Therefore, it is necessary to consider that she has primary ITP associated with hereditary factors. Prior to the onset of this illness, the patient did not exhibit any active bleeding symptoms, such as increased menstrual flow or gum bleeding, and therefore had not received any prior treatment. The patient underwent an open laparotomy and drainage for a left TOA. Due to the combination of ITP and postoperative monitoring, platelet counts were consistently below 50 × 10^9^/L, and coagulation dysfunction was observed. As a result, postoperative complications included incisional hematoma with infection and pelvic hematoma formation. Therefore, the possibility of surgery‐associated ITP was not considered during treatment, resulting in an unsatisfactory prognosis for the patients in the later stages. This issue requires attention. Combining emergency surgery with patients who have ITP poses a significant challenge for clinicians. ITP can lead to severe complications, such as postoperative hemorrhage, and may require platelet transfusions, glucocorticosteroids, and immunoglobulin. These treatments increase the cost of care, decrease quality of life, and have a serious impact on prognosis. Therefore, preventing ITP is of utmost importance. For ITP patients, transfusions of blood products should be administered based on their platelet count level and whether they have anemia or spontaneous bleeding tendencies. Perioperative blood management measures should aim for a target platelet level of more than 30 × 10^9^/L and a hemoglobin concentration of 80 g/L before surgery. This approach can be safely and effectively applied to ITP patients. After surgery, it is important to provide perioperative care to patients and remain vigilant for the possibility of secondary bleeding.

Informed consent has been obtained from the patient according to the consent statement given in the manuscript, and written informed consent has been obtained.

## 4 CONCLUSION

A 25‐year‐old woman with a history of sexual activity and primary ITP underwent emergency surgery for a left TOA. Postoperative complications included incision hematoma with infection and pelvic hematoma formation. The patient was readmitted for further investigation and discharged after receiving platelet transfusions and immunotherapy. Preventing ITP is crucial to ensure fertility and quality of life, and perioperative care should be taken to monitor the possibility of secondary bleeding.

TOAs are a significant consequence of PID. OA and PID are frequently diagnosed in individuals who are sexually active. These conditions often manifest with discomfort in the abdomen and pelvis, discharge from the vagina, unusual vaginal bleeding, as well as feelings of nausea and vomiting. Women diagnosed with TOAs may experience a life‐threatening condition called severe sepsis. It is crucial to promptly and effectively resuscitate these women by immediately administering antibiotics, closely monitoring their condition, and regularly reviewing their progress. It is also important to involve experienced senior clinicians in their care right from the beginning and adopt a multidisciplinary approach, which may include high‐dependency unit or intensive care unit support if necessary. Nevertheless, a significant number of individuals may necessitate surgical intervention as a component of their therapy regimen. It is advisable to promote follow‐up in the office to ensure the remission of symptoms.

ITP is a persistent and diverse disease, and medical treatment is seldom able to provide a complete cure. Experiencing a hemorrhagic event has been associated with unfavorable outcomes and heightened mortality in people with ITP. For emergency situations and severe bleeding in ITP, the goal of treatment should be to quickly increase platelet levels to a sufficient level in order to establish hemostasis. Women diagnosed with ITP may require special consideration regarding their susceptibility to bleeding and blood clotting. The management of patients with ITP is centred around the difficulty of balancing the risks of bleeding and thrombosis. It is personalized and adapted to the individual's risk profile. Accurately categorizing patients based on their bleeding risk would also be advantageous in scenarios involving invasive operations and perioperative care. ITP is a potential hazard of both bleeding and thrombotic occurrences. Additional study is required to enhance knowledge and establish evidence‐based, uniform recommendations, as well as reliable instruments, to assist clinicians in making decisions and managing the trade‐off between the risk of bleeding and thrombosis in patients with ITP.

## AUTHOR CONTRIBUTIONS

**Liqiong Zeng:** Writing – original draft; writing – review and editing. **Libi Tian:** Supervision.

## FUNDING INFORMATION

There are no funders to report in this submission.

## CONFLICT OF INTEREST STATEMENT

The authors declare no conflict of interest.

## ETHICS STATEMENT

All the procedures performed in studies involving human participants were in accordance with the ethical standards of Ethics Committee of University‐Town Hospital of Chongqing Medical University and with the 1964 Helsinki Declaration and its later amendments or comparable ethical standards. The publication of this case report got permission of the patient.

## CONSENT

Written informed consent was obtained from patient to publish this report in accordance with the journal's patient consent policy.

## Data Availability

All data analyzed during the current study are available from the corresponding author upon reasonable request.
